# The Role of ROS as a Double-Edged Sword in (In)Fertility: The Impact of Cancer Treatment

**DOI:** 10.3390/cancers14061585

**Published:** 2022-03-21

**Authors:** Sara Mendes, Rosália Sá, Manuel Magalhães, Franklim Marques, Mário Sousa, Elisabete Silva

**Affiliations:** 1 Department of Physical Education and Sports, University Institute of Maia (ISMAI), 4475-690 Maia, Portugal; saramendes313@gmail.com; 2 Research Center in Sports Sciences, Health Sciences and Human Development (CIDESD), 5001-801 Vila Real, Portugal; 3 Laboratory of Cell Biology, Department of Microscopy, Institute of Biomedical Sciences Abel Salazar (ICBAS), University of Porto, Rua Jorge Viterbo Ferreira, 228, 4050-313 Porto, Portugal; rmsa@icbas.up.pt (R.S.); msousa@icbas.up.pt (M.S.); 4 Unit for Multidisciplinary Research in Biomedicine (UMIB), Laboratory for Integrative and Translational Research in Population Health (ITR), University of Porto, 4099-002 Porto, Portugal; manuel.magalhaes@gmail.com; 5 Department of Oncology, University Hospital Center of Porto (CHUP), Largo do Prof. Abel Salazar, 4099-001 Porto, Portugal; franklim.oncologia@gmail.com; 6 Laboratory of General Physiology, Department of Immuno-Physiology and Pharmacology, Institute of Biomedical Sciences Abel Salazar (ICBAS), University of Porto, Rua Jorge Viterbo Ferreira, 228, 4050-313 Porto, Portugal; 7 Institute for Molecular and Cell Biology (IBMC), Institute for Research & Innovation in Health (I3S), University of Porto, Rua Alfredo Allen, 4200-135 Porto, Portugal

**Keywords:** reactive oxygen species (ROS), oxidative stress, chemotherapy, targeted agents, (in)fertility

## Abstract

**Simple Summary:**

Tumor cells are highly resistant to oxidative stress, but beyond a certain threshold, it may lead to apoptosis/necrosis. Thus, induced loss of redox balance can be a strategy used in anticancer therapies. However, the effectiveness of drugs contrasts with unknown mechanisms involved in the loss of fertility. Considering that cancer patients’ life expectancy is increasing, it raises concerns about the unknown adverse effects. Therefore, new strategies should be pursued alongside explaining to the patients their options regarding the reproduction side effects.

**Abstract:**

Tumor cells are highly resistant to oxidative stress resulting from the imbalance between high reactive oxygen species (ROS) production and insufficient antioxidant defenses. However, when intracellular levels of ROS rise beyond a certain threshold, largely above cancer cells’ capacity to reduce it, they may ultimately lead to apoptosis or necrosis. This is, in fact, one of the molecular mechanisms of anticancer drugs, as most chemotherapeutic treatments alter redox homeostasis by further elevation of intracellular ROS levels or inhibition of antioxidant pathways. In traditional chemotherapy, it is widely accepted that most therapeutic effects are due to ROS-mediated cell damage, but in targeted therapies, ROS-mediated effects are mostly unknown and data are still emerging. The increasing effectiveness of anticancer treatments has raised new challenges, especially in the field of reproduction. With cancer patients’ life expectancy increasing, many aiming to become parents will be confronted with the adverse effects of treatments. Consequently, concerns about the impact of anticancer therapies on reproductive capacity are of particular interest. In this review, we begin with a short introduction on anticancer therapies, then address ROS physiological/pathophysiological roles in both male and female reproductive systems, and finish with ROS-mediated adverse effects of anticancer treatments in reproduction.

## 1. Introduction

Surgery and radiotherapy are commonly used in patients with local and non-metastatic cancer, as they are still the most effective. However, they are very inefficient when cancer is spread throughout the body. In this case, chemotherapeutic drugs or biological agents are among the first-line choice therapies, since they are capable of reaching all organs of the body, through the bloodstream [1].

Chemotherapeutic drugs act primarily by inhibiting the high proliferation rate of cancer cells. One of the molecular mechanisms by which these drugs act is by increasing intracellular levels of reactive oxygen species (ROS) [2] largely beyond cancer cells’ capacity to reduce it, leading to apoptosis or tissue necrosis [3], this has also been observed in the treatment of reproductive cancer [4,5,6]. Treatment-associated excess ROS production in healthy tissues can be the root of cell toxicity observed during chemotherapy treatment, such as anthracycline-mediated cardiotoxicity, and nephrotoxicity triggered by platinum-based compounds [7,8]. Additionally, undesirable side effects on fast renewing cells of the body, such as hair follicles, bone marrow, and gastrointestinal tract cells [1] are also frequently observed.

Biological agents, on the other hand, are more selective molecules that block specific transduction pathways or proteins that are overexpressed/mutated in cancer. The use of biologicals minimizes loss of viability of normal cells and avoids undesirable chemotherapy-associated side effects [1,9]. 

Although targeted therapies were promised to be magic bullets with single targets, the accumulated information obtained through their clinical use has shown side effects of such therapies [10,11,12] and wider mechanisms of action, including ROS-mediated ones [13,14,15].

The increasing effectiveness of anticancer treatments has raised new challenges, especially in the field of reproduction. As the life expectancy of cancer patients has been highly increased, patients who suffered from cancer in their youth will aim to become parents in adulthood and many will be confronted with fertility issues related to side effects of anticancer therapies. In parallel, patients that are living with cancer as a chronic illness through effective therapy may also be faced with this same issue. Therefore, concerns regarding the adverse effects of anticancer therapies on fertility have increased and are of particular interest, namely those impairing the adequate state of gonads and normal sexual functions. As male and female reproductive system(s) and associated gametogenesis, spermatogenesis, and oogenesis, respectively, show a wide range of differences, it is expected to find specific gonadal toxicity for each gender [16,17,18,19,20]. Male fertility dysfunction associated with anticancer therapies can occur by direct gonadotoxic effects and/or disruption of the hypothalamic–pituitary axis, the latest resulting in impaired libido, erection, sperm production, and ejaculation [21]. In females, anticancer drugs can have a direct toxic effect in the ovary and the uterus and cause dysregulation of the hypothalamic-pituitary axis. This may lead to a loss of libido, abnormal follicle development, and impairment of ovarian and uterine function [22,23]. Therefore, in individuals of child-bearing ages, it is necessary to inform them about the possible side-effects on fertility and available fertility preservation options. Post-treatment inability or difficulty to achieve pregnancy is a possible outcome. In fact, chemotherapeutic agents induce numeric or structural chromosomal abnormalities in the germline that, consequently, may impair reproduction by interfering with embryonic development, increasing miscarriages, or transmitting genetic anomalies to offspring [24,25,26,27].

Studies have reviewed the cytotoxic effects of chemotherapeutic drugs [16,18,21,23,24,28,29,30,31,32,33,34,35,36,37,38,39,40], and, to a lesser extent, target therapies [18,28,32,33] on fertility. However, the mechanisms by which they impair fertility are still largely unexplored. This knowledge will be important to define strategies to prevent or attenuate the impact of cancer therapies upon fertility. Thereby, in this review, we address the knowledge on the effects of anticancer treatments (traditional chemotherapy and targeted agents) on fertility, with a special focus on ROS physiological and pathological roles. 

## 2. Oxidative Stress and Fertility

### 2.1. Reactive Oxygen Species and Oxidative Stress

ROS are reactive molecules that contain oxygen atoms and are subdivided into free radicals and non-radical oxidants [41]. ROS reactivity, half-lives, and diffusion capacity are widely variable [42,43,44]. ROS can be generated endogenously by mitochondrial respiratory chain enzymes, nicotinamide adenine dinucleotide phosphate oxidase (NADPH oxidase), microsomal cytochrome P450, or by xanthine oxidase [45]. Beyond endogenous sources, ROS can also have exogenous sources, including ultraviolet radiation, X- and gamma-rays, ultrasound, pesticides, herbicides, and xenobiotics [45].

To cope with the continued production of ROS, cells have developed antioxidant mechanisms that delay or prevent oxidation from happening. In cells, excess ROS are quenched by enzymatic antioxidants such as superoxide dismutase (SOD), catalase (Cat), and glutathione peroxidase (GPx) [46], or nonenzymatic antioxidants such as vitamins A, C, and E, glutathione, and plant polyphenols. Additionally, minerals such as zinc and selenium can have indirect antioxidant effects by acting as cofactors of antioxidant enzymes and other proteins that modulate cellular redox balance [47].

**Figure 1:**
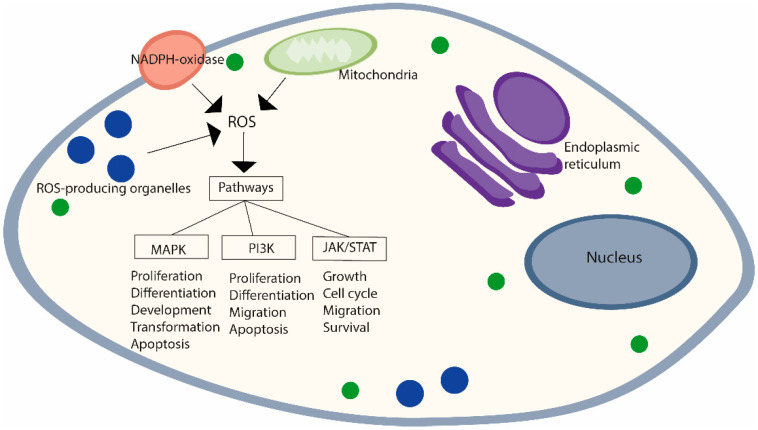
ROS-mediated activation of cell signaling pathways. Major sites of reactive oxygen species (ROS) production in cells, enzymes responsible for ROS production at each of the cellular compartments, and principal signaling pathways activated.

ROS are normal products of cell metabolism with known physiological roles. Even in small amounts, ROS are able to regulate a wide spectrum of signaling pathways, such as the mitogen-activated protein kinase (MAPK), Janus kinase (JAK)/signal transducer and activator of transcription proteins (STATs), and phosphoinositide 3-kinase (PI3K)/protein kinase B (Akt) pathways [48]. They regulate these pathways by modifying the activity of structural proteins, transcription factors, membrane receptors, ion channels, and protein kinases/phosphatases [49]. The MAPK cascade, composed of at least three MAPKs, extracellular signal-regulated kinase (ERK), Janus kinase (JNK/SAPK), and p38 MAPK, plays important roles in cellular processes such as proliferation, differentiation, development, transformation, and apoptosis [50]. The JAK/STAT pathway is used to transduce a multitude of signals. Its activation stimulates cell proliferation, differentiation, migration, and apoptosis [51]. PI3K activation affects several cell events like growth, cell cycle entry, migration, and survival [52] (Figure 1).

Despite ROS having a physiological function, when redox homeostasis is disturbed, due to an imbalance between their production and neutralization, a new state referred to as oxidative stress (OS) may arise. Cells have a graded response to OS. Minor or moderated changes allow cells to adapt and restore redox homeostasis while violent perturbations impair redox signaling, promote biomolecule modifications, and may even induce cell death [53,54].

### 2.2. Reactive Oxygen Species, Oxidative Stress, and Male (In)Fertility 

Spermatogenesis lasts about 70 days, the time needed for the germ stem cell (spermatogonial stem cells) to give rise to the spermatozoon (about 70 million sperm daily), going through mitotic and meiotic divisions [55]. Spermatogonia, the precursors of all germ cell types, are located or “resting” on the basal lamina (a modified form of extracellular matrix (ECM) constituted by collagen and myoid cell layers). Basal lamina plays a significant role in spermatogenesis. It behaves as a physical support to the seminiferous epithelium, provides selected access of molecules to the seminiferous epithelium, and enables the crosstalk between the seminiferous epithelium, myoid cells, and interstitial cells, such as Leydig cells [56]. Spermatogonia present a continuous self-renewing capacity and are responsible for making a balance between germ and Sertoli cells [57].

**Figure 2:**
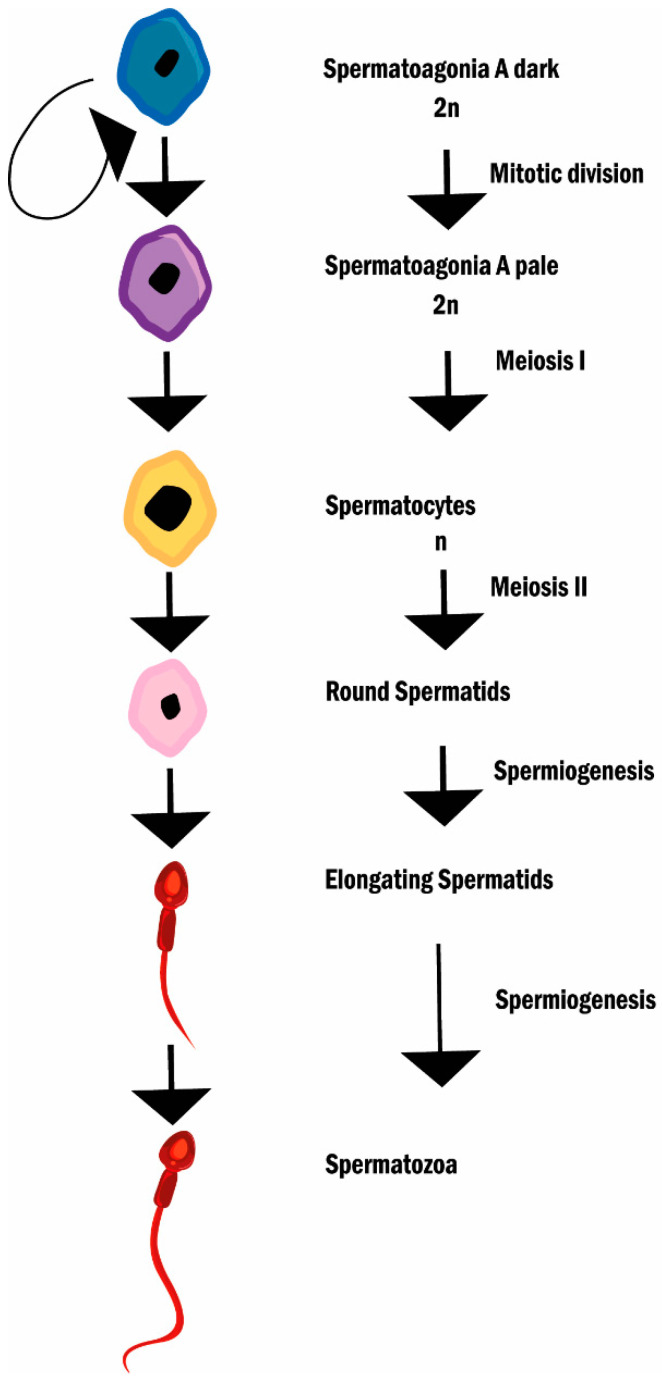
Spermatogenesis and spermiogenesis. The diagram describes the different stages of spermatogenesis and spermiogenesis.

Spermatogonia A dark (SGAd) divides into spermatogonia A pale (SGAp) and SGAd thus, maintaining the stem cell pool. SGAp then divides to give origin to spermatogonia B and these to pre-leptotene spermatocytes. Pre-leptotene spermatocytes (primary spermatocytes) now enter meiosis and progressively turn into zygotene spermatocytes, and then into pachytene spermatocytes [58]. At this stage, the cell attains the biggest volume and starts crossing over (chromosome recombination). Primary spermatocytes then complete the first meiotic division with separation of chromosomes (not of chromatids as in mitosis) and give rise to secondary spermatocytes, that go through the second division (separation of chromatids) and originate haploid round spermatids. Spermatids progressively differentiate into spermatozoa [59], the nucleus condenses and elongates, and a flagellum is formed (spermiogenesis). The condensation of the nucleus is due to the substitution of histones by protamines, a basic protein that establishes bisulfite links enabling the compaction of DNA in order to protect against mechanical and oxidative stresses that sperm will encounter during the journey until the Fallopian tube where fertilization occurs [60]. From the Golgi, a series of vesicles fuse and give rise to an acrosomal vesicle that surrounds the anterior 2/3 of the nucleus. This vesicle contains the enzymes necessary to aid in the penetration of the zona pellucida [61]. When these steps finalize, the sperm retracts from Sertoli cell junctions (spermiation) and travels through the tubules of the rete testis to the epididymis where it matures and remains stored up to ejaculation [62] (Figure 2).

During human spermatozoa maturation, differentiated levels of ROS can be produced by plasmatic membrane NADPH oxidase and by mitochondrial nicotinamide adenine dinucleotide-dependent oxidoreductase [63,64], being highest in immature spermatozoa with abnormal head morphology and cytoplasmic retention and lowest in mature spermatozoa and immature germ cells [65,66]. Moreover, spermatogenesis appears to be paralleled by a differential expression of stress response genes, as suggested by an increase in the antioxidant enzyme Cu-Zn SOD [67]. This points to changes in susceptibility to OS through spermatogenesis, which can be rooted in distinct reasons. Late stages of spermatids and spermatozoa are vulnerable to the deleterious effects of ROS due to high levels of polyunsaturated fatty acids in the plasma membrane (essential for membrane fluidity), whereas differentiating spermatogonia and spermatocytes appear susceptible due to their high mitotic and meiotic activity, respectively [68,69]. ROS have a pivotal role in spermatogenesis, not only in the earliest stages of development, being involved in sperm chromatin condensation, in inducing apoptosis to adjust the number of germ cells or spermatogonia proliferation, but also in sperm maturation, participating in capacitation, acrosome reaction, mitochondrial sheath stability, and sperm motility [70,71,72,73]. 

ROS are also implicated in sperm-oocyte interaction [74] and participate in the activation of the steroidogenic pathway by inducing Ras and ERK1/2 activation in Leydig cells [75]. In the seminal fluid, leukocytes produce high levels of ROS (up to 1000 times more than spermatozoa) that play an important role in the cellular defense mechanism against infections and inflammation [76].

The pathophysiological role of ROS in spermatogenesis has also been studied. Intrinsic ROS overproduction depletes sperm antioxidant systems, leading to OS [77,78]. OS impinges on molecular components, inducing oxidation in lipids, proteins, carbohydrates, and DNA. Oxidative modifications to proteins alter their structure and function, with repercussions both on spermatogenesis and fertility [79]. Peroxidative damage to membrane lipids leads to membrane structure and fluidity instability and membrane-associated processes dysregulation [79,80]. Even sperm motility is affected by lipid peroxidation to mitochondrial membranes, leading to a decrease in mitochondrial membrane potential and defects in the sperm mid-piece and axonemal region [81]. OS also has deleterious effects on the spermatic nucleus, impinging on DNA integrity, increasing the rates of sperm DNA fragmentation [73,74,82]. Since spermatozoa lack DNA repair mechanisms, in case of excessive DNA damage apoptotic cascades are activated, leading to reduced sperm concentrations and consequently male infertility [83]. Recently, it has been observed that about 30–80% of infertile men have abnormal semen characteristics with elevated seminal ROS levels [84].

### 2.3. Reactive Oxygen Species, Oxidative Stress, and Female (In)Fertility

During early embryo development, primordial germ cells migrate to the developing gonads, undergo mitotic divisions (before entering meiosis), colonizing it. Primordial germ cells become oogonia and rearrange themselves in structures described as germ-cell nests. After entering meiosis, individual oocytes are encapsulated by a single layer of squamous pre-granulosa cells, forming primordial follicles [85]. After a programmed cell nest breakdown, only around 30% of the initial oogonia survive and become the pool of primordial follicles. Oocytes remain arrested in the first meiotic division until latter activation to proceed development [86]. Early activation, during childhood and until puberty, results in atresia (degeneration of follicle into scar tissue). For this reason, in puberty, ~300,000 primordial follicles remain [87,88]. Of this pool, as little as 400 follicles will complete development and ovulate; all the others will suffer atresia [89,90].

**Figure 3:**
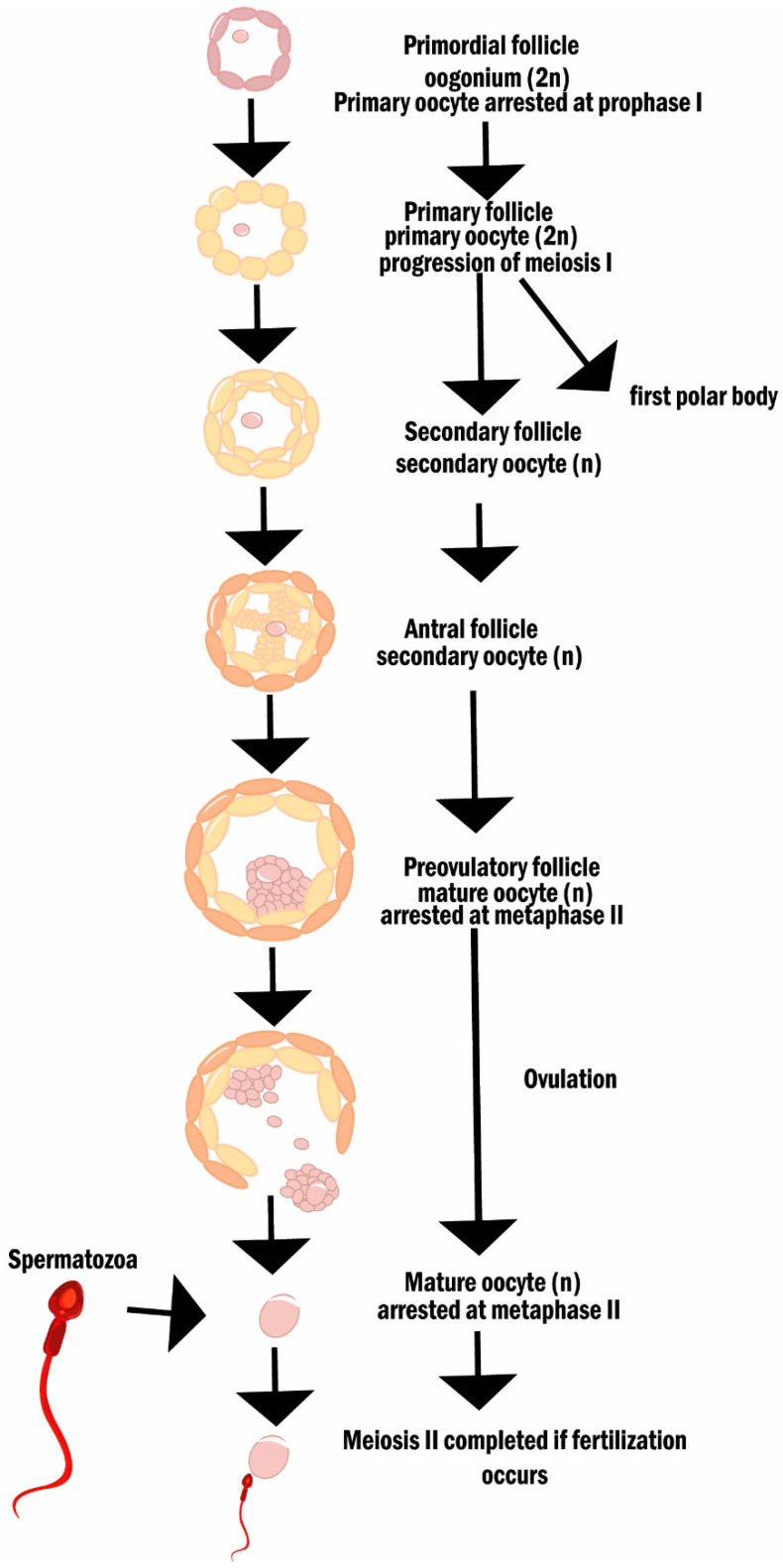
Oogenesis and folliculogenesis. The diagram describes the different stages of oogenesis and folliculogenesis.

From puberty, and until menopause, hormones produced by the hypothalamus, pituitary gland, and the ovaries are the messengers responsible for the ovarian cycle, which can be divided into two phases: follicular and luteal. The follicular phase concerns the development of follicles until ovulation. In short, once a selected batch of primordial follicles is activated, their granulosa cells change shape and give rise to primary follicles. These follicles express cell proliferation markers which will allow their growth and ultimately the formation of a multi-layered follicle—the secondary follicle. The antral stage follows and is achieved by the formation of a cavity (the antrum) filled with fluid. In humans, only one dominant follicle will develop, until ovulation. The ovulated oocyte, arrested at metaphase I, will complete meiosis only if fertilization occurs (Figure 3).

The luteal phase starts after ovulation, with the formation of the *corpus luteum*, and is characterized by changes in hormone levels (an increase in progesterone and in estrogen and a decrease in follicle-stimulating hormone (FSH) and in luteinizing hormone). These hormonal fluctuations will regulate uterine transformations to enable implantation [91]. Upon fertilization, the zygote moves through the fallopian tube until reaching the uterine endometrium, where implantation may take place [92]. If a pregnancy does not occur, hormone production by *corpus luteum* declines, causing endometrial shedding, and marking the end of the luteal phase [91,93,94,95]. For a more detailed description see [91].

In the ovaries, ROS can be generated by macrophages, steroidogenic cells, and endothelial cells, modulating follicular fluid microenvironment and consequently oocyte development [96]. ROS are involved in the loss of sensitivity of granulosa cells to gonadotropins and steroidogenic function, thus influencing follicular atresia and having a role in the selection of the dominant follicle [97,98]. In the pre-ovulatory follicles, steroid production increases cytochrome P450 activity and consequently the levels of ROS, which are important inducers of ovulation. In fact, decreased ROS production impairs ovulation [99,100]. During oocyte maturation, the expression of enzymatic antioxidants such as Cu-ZnSOD and MnSOD revealed that oocytes are exposed to high levels of ROS and that the balance between ROS and antioxidant enzymes is an important modulator of this process [101]. Although ROS have important physiological roles, the cyclic production of these damaging agents over time and a reduction in ovarian antioxidant capacity may be the root of local inflammation and fibrosis and contribute to tissue dysfunction and the loss of fertility [102,103,104].

In the uterus, ROS have also been implicated in the regulation of the endometrial cycle alongside variations in the expression of SOD, GPx, and lipid peroxides (in response to sex hormones) [105,106,107]. NADPH-oxidase-derived O_2_ has been shown to activate the nuclear factor kappa-light-chain-enhancer of activated B cells (NF-kB) signaling cascade promoting prostaglandin secretion, vasoconstriction, and ultimately endometrial shedding [108,109]. Thus, ROS have a determinant role in the regulation of angiogenesis and the endometrial cycle [110,111]. NF-kB exacerbated activation, due to increased uterine levels of ROS, may result in signaling pathways disruption and, consequently, in a broad spectrum of uterine-related infertility disorders (e.g., endometriosis) [112,113].

ROS play a bivalent role (physiological and pathophysiological) not only in the uterus and the ovaries but also in the process of placentation, as previously reviewed [114].

## 3. Current Evidence of OS-Mediated Effects on Fertility Derived from Cancer Therapies

### 3.1. Carcinogenesis, Anticancer Therapies and Oxidative Stress

Low-to-moderate ROS levels act as instigators of neoplastic transformation, by promoting genomic DNA mutations and increasing cell proliferation [115,116]. After neoplastic transformation, during hyperproliferation, cancer cells present uncontrolled metabolism and high basal levels of ROS [117]. Their survival under such adverse conditions is achieved due to antioxidant system adaptations [118]. However, if ROS levels increase above a certain threshold (even in neoplastic cells), it will lead to antioxidant system exhaustion and evoke irreversible oxidative damage. The majority of agents used in anticancer therapies aim to induce an accelerated and cumulative oxidative damage, which will surpass the cytotoxic threshold and “selectively” kill cancer cells [119].

There are two major approaches of eliciting intracellular ROS accumulation that are harnessed by anticancer therapies: direct ROS generation or cellular antioxidant system inhibition [120] (Table 1).

ROS-promoting agents can: increase the production of O_2_ by impairing respiratory chain function and causing mitochondrial dysfunction [130] or by activating NADPH-oxidase activity [131]; increase radical intermediates by reacting with flavoprotein reductases (e.g., cytochrome P450 reductase) in the presence of reduced NADPH [132]; lead to hydroxyl radical formation by triggering Fenton-type reactions [133]. Doxorubicin promotes an increase in ROS by intracellular chelation of iron, which may trigger a Fenton-like reaction, generating the high reactive hydroxyl radical, and by interfering with cytochrome P450 forming radical derivatives, which can generate superoxide, in the presence of oxygen [134].

Agents that strategically interfere with ROS metabolism by inducing the depletion of the reduced glutathione (GSH) pool or restricting redox modulating enzymes (e.g., peroxidases and peroxidoxins) have a profound effect on the ability of cells to detoxify ROS. GSH-conjugating compounds, such as Imexon [135] or targeting its synthesis, such as Buthionine sulphoximine, fall into this group [128]. Specific inhibitors of other antioxidant enzymes are being identified, developed, and used in anticancer treatments. ATN-224, an inhibitor of SOD [136,137], and AT, an inhibitor of Cat [138], fall into this category.

Understanding the distinct mechanisms of action of each drug, either ROS-dependent or -independent is vital to attenuate their deleterious side effects. In fact, cotreatments with antioxidants have been used to ameliorate chemotherapy-mediated toxicity (e.g., nephrotoxicity and ototoxicity). Kilic and colleagues have demonstrated that cotreatment with melatonin significantly reduces NF-kB expression and is able to attenuate nephrotoxicity through the activation of nuclear factor-erythroid factor 2-related factor 2 (Nrf-2)/Heme oxygenase-1 pathway [139]. This pathway regulates the expression of several antioxidant genes and protects against OS and inflammation [139,140]. Additionally in the specific context of chemotherapeutical treatments, flavonoids and carotenoids (plant-derived phytochemicals) have been shown to have beneficial properties against ROS-associated secondary effects [141,142,143].

### 3.2. Treatment-Induced OS and Its Impact on Fertility

Although anticancer therapies aim to specifically disrupt the redox balance of cancer cells, unwanted effects on normal cells also occur. Encephalo-, cardio-, nephron-, oto-, hepato-, myeolo-, myo-, and gastrointestinal toxicities have been described [144,145,146,147,148]. Similarly, distinct gonad toxicity has also been reported [149,150], reflecting the differences between the two gametogenesis processes. 

**Figure 4:**
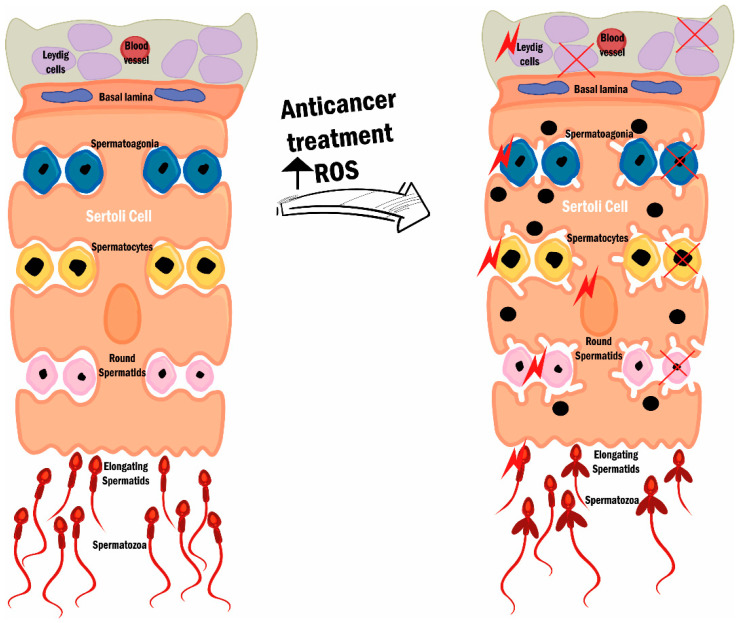
Spermatogenesis dysfunction after anticancer treatment. ROS overproduction due to treatments depletes the antioxidant systems, leading to OS. Both the normal and abnormal spermatozoa can be damaged by ROS; however, in the treatment case (right side), damage is more prevalent since ROS are present/produced in higher quantity due to anticancer treatments. OS impinges on spermatozoa (represented by the red stars) and damages to cell/sperm and mitochondria membranes, DNA damage, and defects in the sperm mid-piece and axonemal region can be observed. The establishment of this compromised process leads to abnormal semen characteristics and is responsible for the fertility decline present in men submitted to anticancer treatments. Reactive oxygen species (ROS).

In males, depending on the type of spermatogonia affected, damage can result in transient or persistent oligozoospermia or azoospermia. Spermatogonia type B are more susceptible to cytotoxicity because of their active mitotic proliferation, whereas spermatogonia stem cells (type A) are less susceptible due to their low mitotic activity [16]. In severe gonad toxicity, all spermatogonia are destroyed and azoospermia is established. Additionally, even Sertoli or Leydig cells can be damaged. Leydig cell damage also affects hormone production [151,152,153] (Figure 4).

**Figure 5:**
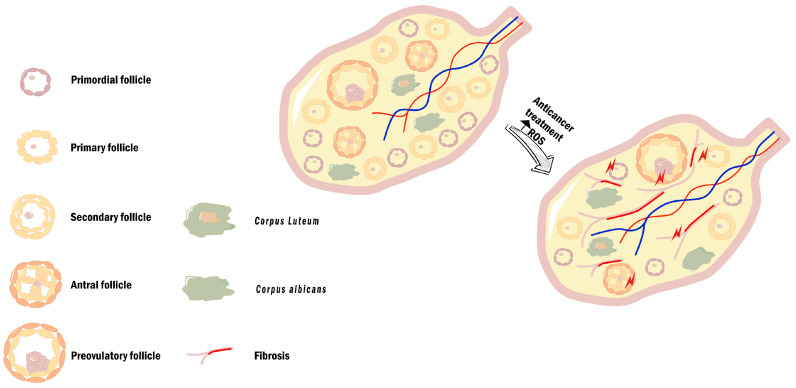
Ovarian tissue dysfunction after anticancer treatments. Increase in OS-derived from anticancer treatment, due to increased ROS production and impaired antioxidant response leads to the establishment of an oxidative microenvironment. In a post-treatment ovarian stroma, a depletion in the number of primordial and primary follicles and the presence of collagen deposition can be observed (fibrosis). The establishment of this compromised microenvironment impairs ovarian function and is responsible for the fertility decline present in women submitted to anticancer treatments. Reactive oxygen species (ROS). Cisplatin and doxorubicin are two widely used chemotherapeutic drugs to treat several types of cancer, including those of the reproductive tract. Their ROS-mediated effects on fertility will now be revised.

Females, unlike males, have a limited reproductive life span that is dependent on the number of primordial follicles (see Section 2.3 of this review). As such, conventional chemotherapeutic agents can lead to permanent ovarian failure and amenorrhea due to oocyte depletion [154,155]. This can occur by direct damage to granulosa cells, as these cells are an easy target for chemotherapeutic agents due to their highly proliferative rate. Reduced number of granulosa cells might deprive the oocyte of nutrient supply and disrupt granulosa/oocyte communication (vital for oocyte maturation), inducing oocyte apoptosis [156]. Even when gametes are spared, it is still possible that the damage caused to other ovarian components, such as ovarian vasculature and stroma, will also contribute to premature ovarian failure [157,158,159] (Figure 5).

Cisplatin is a highly reactive molecule that binds to DNA and forms nDNA (nuclear DNA) adducts (mechanism of cytotoxicity) and mitochondrial DNA (mtDNA) adducts (ROS-promoting mechanism). Cisplatin interacts with DNA by mainly forming Pt-d (GpG) di-adducts, which if not repaired by the DNA damage response will block replication and/or transcription and lead to apoptosis [123,160]. Cisplatin also binds to RNA and proteins. Mitochondrial membrane proteins, particularly voltage-dependent anion channels, are preferential binding sites [161]. Cisplatin also interferes with the activity of several proteins involved in the maintenance of redox balance. In the testicular tissue, cisplatin decreases GSH and Cat activity, which may increase the vulnerability of germ cells to ROS deleterious effects [162,163]. An increase in cisplatin-mediated ROS, at the testicular ECM, activates fibroblasts (by transforming growth factor-beta upregulation), and increases collagen accumulation with deleterious consequences in the structure of the seminiferous epithelium and a reduction in the spermatogenic activity [162,164]. In Leydig cells, dysfunction was also observed, as cisplatin exerts an inhibitory action at the level of cytochrome P450, inhibiting testosterone synthesis [165,166]. Broader cisplatin damage on spermatogenic parameters includes abnormalities in sperm motility and sperm morphology [167]. In the ovaries, cisplatin increases primordial follicle activation and granulosa cells apoptosis, leading to primordial follicles depletion [168,169]. It also increases the end-product of lipid peroxidation, malondialdehyde (MDA), and decreases SOD and GSH antioxidant activity. Chemoprotective effects have been observed with the use of molecules with antioxidant properties [170,171,172].

Doxorubicin, an antiproliferative (by inhibition of topoisomerase II), highly reactive (by DNA intercalation), and ROS promotor (by iron chelation) molecule, does not exclusively damage neoplastic cells, but also healthy dividing cells, as germ cells. In males, unwanted reproductive side effects occur through an increase in testicular oxidative stress, inflammation, and apoptosis [173,174,175]. A reduction in sperm quality (e.g., loss of acrosome integrity or morphological abnormalities, and motility) has been seen after doxorubicin treatment [176,177]. A decrease in body and relative testicular weights, reduced seminiferous tubule diameter, and germinal epithelium thickness have also been observed [178]. These changes probably result from atrophy of Leydig cells and the reduction in the germ cell number and spermatogenic proliferative rate [179,180]. Additionally, in the testicular tissue of doxorubicin-treated subjects, the levels of apoptotic-related genes (e.g., caspase 3 and B-cell lymphoma 2 genes) and MDA are increased, and the activities of antioxidant enzymes (SOD and GPx) are reduced [176,179]. In female mice, doxorubicin administration induced an imbalance in the redox state, by interfering with the activation of the antioxidant Nrf-2 pathway and the expression of antioxidant enzymes SOD, Cat, and GPx [181,182]. Divergent results regarding the expression of antioxidant enzymes in response to doxorubicin treatment have been reported and may result from experimental designs used and the specific self-protective response of cells (oocytes, granulosa, and cumulus) [181,182]. Nevertheless, beneficial effects were observed with the use of molecules that modulate redox balance [181,182,183]. Just like in males, doxorubicin influences the inflammatory response by inducing a significant increase in the expression of pro-inflammatory cytokines (e.g., tumor necrosis factor-alpha, interleukin 6 and 8) [184]. Inflammation activates matrix metalloproteinases (MMPs) and induces alterations in the degradation of the extracellular matrix that may favor an excessive collagen deposition and contribute to ovarian fibrosis [185]. Additionally, MMPs activation may also regulate the local recruitment and availability of inflammatory mediators, acting as a positive feedback loop of inflammation. Doxorubicin treatment is associated with local inflammatory responses and morphological damage to oocytes and stroma, and protective effects of antioxidant molecules were, once more, observed [184].

Although studies on the effects of molecular target therapy on fertility are still scarce, some data on male fertility are available [186,187]. Bortezomib, an antineoplastic agent and a proteasome inhibitor frequently used for multiple myeloma and mantle cell lymphoma, induces tumor cell apoptosis via the induction of endoplasmic reticulum stress (the capacity to fold proteins becomes saturated), increased expression of p53 (tumor suppressor), and activation of caspase-3 (cell death inductors) [188]. In males, bortezomib induces germ cell development arrest, impairing the spermatogenic process [189]. In fact, a study by Li and colleagues demonstrated that OS induced by bortezomib increased testicular MDA and decreased GPx and total SOD protein levels [190]. Besides, it also caused an imbalance in cell signaling, disrupted Sertoli-germ cell anchoring junctions, and interfered with spermatogenesis. Additionally, the study also provided evidence that FSH counteracted bortezomib’s negative effects by regulation of a pro-survival response to OS-mediated insults to Sertoli cells (via Akt/ERK pathway) [190].

Other less investigated anticarcinogenic agents, their ROS-dependent mechanisms of action, and effects on fertility are summarized in Table 2.

## 4. Conclusions

Induced loss of redox balance can be a strategy used in anticancer therapies. However, the effectiveness of drugs contrasts with new problems and challenges that arise from the increase in patients’ survival and their aims to become parents. As ROS have a pivotal role in male and female gametogenesis processes, ROS-associated side effects of anticancer therapies on reproductive systems can compromise fertility. For this reason, there has been an increase in studies aiming to shed light on the mechanisms involved in the loss of fertility associated with anticancer treatments and innovative ways of ameliorating them. 

It is important to continue the pursuit of such new strategies and in parallel explain to patients the available options to bypass anticancer treatment side effects on the reproductive system.

## Figures and Tables

**Table 1:** Major anticancer compounds and respective ROS-mediated actions.

Name	Mechanism of Action	Role in Redox System	Ref
Direct ROS generation			
5-fluorouracil	Thymidylate synthase inhibitor	p53-dependent ROS	[121]
Bortezomib	Proteasome inhibitor	ER stress-induced ROS	[122]
Cisplatin	nDNA adducts generation	mtDNA and ETC damage	[123]
Doxorubicin	nDNA intercalation; topoisomerase-II-mediated nDNA repair disruption	Generation of free radical through iron chelation	[124]
Erlotinib	EGFR tyrosine kinase inhibition	Loss of MM potential	[125]
Imatinib	Bcr-Abl tyrosine kinase inhibition	Loss of MM potential	[126]
Rituximab	Anti-CD20	Bcl-2 inhibition	[127]
Antioxidant process inhibition			
Buthionine sulfoximine	-	GSH synthesis inhibitor	[128]
Imexon	Ribonucleotide reductase inhibitor	GSH activity disruption via thiol binding	[129]

Abelson (Abl); B-cell lymphoma 2 (Bcl-2); breakpoint cluster region protein (Bcr); cluster of differentiate 20 (CD20); electron transport chain (ETC); endoplasmic reticulum (ER); epidermal growth factor receptor (EGFR); glutathione (GSH); mitochondrial DNA (mtDNA); mitochondrial membrane (MM); nuclear DNA (nDNA); reactive oxygen species (ROS).

**Table 2:** Other anticarcinogenic agents and their effects on fertility.

Name	Fertility Effect	Ref	ROS-Known Effect	Ref
5-fluorouracil	Decreased sperm count (rat)	[191]	Inflammation, autophagy, apoptosis, and senescence induction	[192,193,194]
Erlotinib	-		Increase radical’s production through NOX4	[195]
Imatinib	Reduces sperm count and density (human)	[196]	Reduces MMP and complex I activity of ETC, leading to mitochondrial OS	[197]
	Decrease vasculature of placenta (mouse)	[198]		
	Diminishes primordial follicles (mouse)	[198]		
Rituximab	No mentionable effects (human and mouse)	[199,200,201]	-	
Buthionine sulfoximine	-		Mitochondrial impairment	[202,203]
Imexon	-		GSH depletion and induction of ER stress	[129,135]

Electron transport chain (ETC); endoplasmic reticulum (ER); glutathione (GSH); metalloproteinases (MMP); NADPH oxidase 4 (NOX4); oxidative stress (OS).
